# Concordance between Preoperative mpMRI and Pathological Stage and Its Influence on Nerve-Sparing Surgery in Patients with High-Risk Prostate Cancer

**DOI:** 10.3390/curroncol29040193

**Published:** 2022-03-28

**Authors:** Clara Humke, Benedikt Hoeh, Felix Preisser, Mike Wenzel, Maria N. Welte, Lena Theissen, Boris Bodelle, Jens Koellermann, Thomas Steuber, Alexander Haese, Frederik Roos, Luis Alex Kluth, Andreas Becker, Felix K. H. Chun, Philipp Mandel

**Affiliations:** 1 Department of Urology, University Hospital Frankfurt, Goethe University Frankfurt am Main, 60590 Frankfurt, Germany; felix.preisser@kgu.de (F.P.); mike.wenzel@kgu.de (M.W.); maria.welte@kgu.de (M.N.W.); lena.theissen@kgu.de (L.T.); frederik.roos@medicum-wiesbaden.de (F.R.); luis.kluth@kgu.de (L.A.K.); andreas.becker@kgu.de (A.B.); felix.chun@kgu.de (F.K.H.C.); philipp.mandel@kgu.de (P.M.); 2 Cancer Prognostics and Health Outcomes Unit, Division of Urology, University of Montréal Health Center, Montréal, QC H4A 3J1, Canada; 3 Department of Radiology, University Hospital Frankfurt, Goethe University Frankfurt am Main, 60590 Frankfurt, Germany; boris.bodelle@kgu.de; 4 Dr. Senckenberg Institute of Pathology, University Hospital Frankfurt, 60590 Frankfurt, Germany; jens.koellermann@kgu.de; 5 Martini-Clinic Prostate Cancer Center, University Hospital Hamburg-Eppendorf, 20251 Hamburg, Germany; thomas.steuber@uke.de (T.S.); alexander.haese@uke.de (A.H.)

**Keywords:** prostate cancer, nerve-sparing surgery, radical prostatectomy, multiparametric magnetic resonance imaging

## Abstract

Background: We aimed to determine the concordance between the radiologic stage (rT), using multiparametric magnetic resonance imaging (mpMRI), and pathologic stage (pT) in patients with high-risk prostate cancer and its influence on nerve-sparing surgery compared to the use of the intraoperative frozen section technique (IFST). Methods: The concordance between rT and pT and the rates of nerve-sparing surgery and positive surgical margin were assessed for patients with high-risk prostate cancer who underwent radical prostatectomy. Results: The concordance between the rT and pT stages was shown in 66.4% (*n* = 77) of patients with clinical high-risk prostate cancer. The detection of patients with extraprostatic disease (≥pT3) by preoperative mpMRI showed a sensitivity, negative predictive value and accuracy of 65.1%, 51.7% and 67.5%. In addition to the suspicion of extraprostatic disease in mpMRI (≥rT3), 84.5% (*n* = 56) of patients with ≥rT3 underwent primary nerve-sparing surgery with IFST, resulting in 94.7% (*n* = 54) of men with at least unilateral nerve-sparing surgery after secondary resection with a positive surgical margin rate related to an IFST of 1.8% (*n* = 1). Conclusion: Patients with rT3 should not be immediately excluded from nerve-sparing surgery, as by using IFST some of these patients can safely undergo nerve-sparing surgery.

## 1. Introduction

Prostate cancer (PCa) is the second leading cause of cancer-related mortality and the most commonly diagnosed malignancy among Western men [1]. Historically, the risk classification of PCa is based on a combination of clinical staging (cT), achieved by digital rectal examination (DRE), serum prostate-specific antigen (PSA), and biopsy Gleason score [2]. Thereby, the correct distinction between organ-confined disease (T1–2) and locally advanced stage (T3–4) has a large impact not only on the prognosis, but also on the treatment planning, e.g., for the decision to undergo nerve-sparing surgery (NSS) [3].

In addition to oncologic outcomes, optimal functional outcomes, such as continence and potency, are important for patients undergoing radical prostatectomy (RP) for PCa. Functional outcomes can be improved by NSS [4,5,6]. As patients with high-risk PCa have an increased risk for extracapsular extension (ECE) and/or seminal vesicle invasion (SVI), the preoperative decision on whether to perform NSS or not can be difficult [7]. The EAU guidelines recommend avoiding NSS when there is a risk of ECE, whereby a recent study of Preisser et al. showed that NSS in patients with high-risk PCa was not associated with a worse oncological outcome [3,8]. Therefore, there is a high demand for accurate localization and information on tumour extent prior to surgery, as NSS should not compromise cancer control [9]. So far, mostly DRE or nomograms (e.g., Steuber et al.) have been used to predict ECE, both bearing a high uncertainty in the correct estimation of ECE [10,11,12,13,14,15]. In contrast, multiparametric magnetic resonance imaging (mpMRI) has currently appeared to be a promising method to improve the determination of tumour extent, especially when using it for nomograms [12,13,14]. Additionally, the EAU guidelines state that mpMRI can be useful for treatment planning for locally advanced tumours and guide the decision for nerve-sparing surgery [3,16]. The literature reports wide ranges of sensitivity and specificity with regard to its ability to predict ECE and SVI [12,15,17,18]. Consequently, the capability and clinical value of mpMRI to detect ECE and SVI is still a matter of debate. Therefore, the aim of our study was to assess the concordance between the radiologic stage, determined by mpMRI, and pathological stage in a cohort of men at high risk of ECE. Specifically, we assessed the predictive accuracy of preoperative mpMRI for ECE and its influence for the decision for NSS in patients with high-risk PCa.

## 2. Patients and Methods

### 2.1. Study Population

Data on 160 consecutive patients with high-risk PCa (according to the D’Amico risk classification), who underwent open or robotic RP between January 2018 and June 2020 at the department of Urology, University Hospital Frankfurt, were prospectively collected. From 1 January 2018, all patients with high-risk PCa routinely underwent mpMRI before open or robotically assisted RP. Moreover, for all patients the risk of ECE was calculated according the nomogram of Steuber et al. [11].

Due to uncertainties in pathologic evaluation in patients with neoadjuvant therapy, those patients (*n* = 34) with neoadjuvant androgen deprivation therapy and patients (*n* = 10) with neoadjuvant chemotherapy were excluded from the analysis, resulting in 126 consecutive patients with high-risk PCa who have been included in the current analyses.

The study was approved by the Institutional Review Board (UCT-Projekt-Nr.: SUG-2-2018) and written informed consent was obtained from all patients. The approval date was on 29 July 2020.

### 2.2. mpMRI Protocol and Analysis

mpMRI of the prostate was either performed during the diagnostic workup or for staging purposes. mpMRI included at least T1-weighted, T2-weighted, diffusion-weighed, and dynamic contrast-enhanced imaging and were either performed at outpatient clinics or within our radiologic department. Final radiologic staging (rT) was based on the TNM classification considering the MRI report of the radiologist describing the extracapsular extension with involvement of the neurovascular bundle, seminal invasion, rectal or vesical invasion [3].

### 2.3. Surgical Approach and Pathologic Analysis

The majority of patients underwent bilateral nerve sparing with the intraoperative frozen section technique (IFST) as previously described [19,20]. In very rare cases, primary NSS was not possible due to local tumour spread.

During RP, the tissue of the prostate adjacent to the neurovascular bundle (NVB) was dissected for frozen section. For anatomic orientation, the inner and outer surgical margins, as well as the apical side, were inked with different colours. In the Department of Pathology, the specimens were cut into 3–4 mm-thick slices and all tissue blocks were embedded in freezing media. Stained in haematoxylin and eosin, six µm cryosections were cut from each block to be reviewed by a dedicated uropathologist. At least one malignant gland should have come into contact with the inked surgical margin to be determined as a positive surgical margin (PSM) [21]. In case of PSM in the area of NVB during frozen section, a subsequent resection of the corresponding NVB was performed.

### 2.4. Statistical Analysis

Descriptive statistics included frequencies and proportions for categorical coded variables. Medians and interquartile ranges (IQR) were reported for continuously coded variables. Univariable analysis were performed to compare concordance of tumour stage based on preoperative MRI characteristics (rT) with subsequent pathological stage (pT). Specificity, sensitivity, positive predictive value (PPV) and negative predictive value (NPV) and accuracy of mpMRI in predicting extraprostatic disease were calculated. The effect of radiological extraprostatic disease findings on surgical margin rates after RP was examined by comparing PSM between patients with rT2 or ≥rT3. The statistical software GraphPad Prism 5.02 was used (GraphPad Software, San Diego, CA, USA).

## 3. Results

### 3.1. Patients and Preoperative Characteristics

Overall, of all 126 patient, 23.8% (*n* = 30) had a preoperative PSA ≥20 ng/mL, 78.6% (*n* = 99) had a biopsy Gleason score ≥8 and 33.3% (*n* = 42) a suspicious bilateral DRE finding ≥cT2c. Median BMI was 26.5 kg/m^2^ and median age was 66.0 years. Surgery was performed either with the use of an open (*n* = 86, 68.3%) or robotically assisted approach (*n* = 40, 31.7%) (Table 1).

### 3.2. Prediction for Pathologic ECE by Nomograms

According to the nomogram of Steuber et al., the risk of ECE was predicted with means of 55.9% and 72.8% for patients with rT2 and ≥rT3, respectively. When comparing the nomogram to pT-stages, the risk of ECE was predicted with a mean of 60.1% for patients with a subsequent pT2 stage, with 71.8% for patients with ≥pT3.

### 3.3. Comparison of mpMRI and pT-Stage

In total, in the preoperative mpMRI, 47.6% (*n* = 60) of men were classified as rT2, 30.1% (*n* = 38) rT3a, 21.4% (*n* = 27) rT3b and 0.9% (*n* = 1) a rT4 (with suspicion of rectal infiltration). No pathologic lymph nodes were seen in 88.1% (*n* = 111) of patients, whereas 11.9% (*n* = 15) were suspected to have positive lymph nodes. In the histopathological examination of the specimen, 34.1% (*n* = 43) had a pT2, 38.9% (*n* = 49) a pT3a, 23.8% (*n* = 30) a pT3b and 3.2% (*n* = 4) a pT4 stage. 78.6% (*n* = 99) were lymph node negative (pN0), whereas 19.8% (*n* = 25) were lymph node positive (pN1) and 1.6% (*n* = 2) were classified as pNx, respectively (Table 1). Analysing the concordance rate of rT and pT for locally advanced tumour (≥pT3a), the sensitivity specificity, PPV, NPV and accuracy for mpMRI were 65.1%, 72.1%, 81.8%, 51.7% and 67.5%, respectively (Table 2).

### 3.4. Nerve-Sparing Surgery and Positive Surgical Margin

In total, 90.0% (*n* = 54) and 1.7% (*n* = 1) of patients with rT2 underwent primary bilateral or unilateral NSS with IFST. Bilateral, unilateral and no nerve sparing—after secondary resection if necessary—were performed in 68.3% (*n* = 41), 11.7% (*n* = 13) and 10.0% (*n* = 6). of these cases. In total, 25.0% (*n* = 15) of patients with rT2 harboured PSM, whereas 75.0% (*n* = 45) had negative surgical margins.

Despite the morphological suspicion of extraprostatic disease (≥rT3) in mpMRI, 72.7% (*n* = 48) and 12.1% (*n* = 8) of the patients underwent primary bilateral or unilateral NSS with IFST. After secondary resection, bilateral and unilateral NSS was still possible in 42.9% (*n* = 24) and 51.8% (*n* = 29) of those men with ≥rT3, whereas in 5.3% (*n* = 3) no nerves could be spared. A total of 50% (*n* = 28) of the patients with ≥rT3 and primary NSS harboured PSM, while 80% of the patients with ≥rT3 and primary non-NSS had PSM (Table 3).

**Figure 1:**
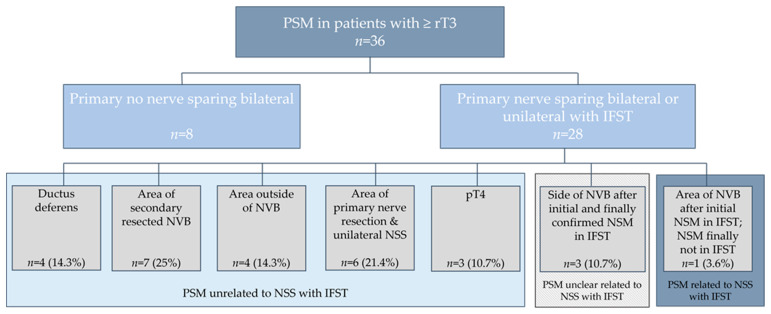
PSM in patients with ≥rT3 & details about area of PSM after nerve sparing surgery (NSS) with IFST. For further analyses patients with primary no nerve sparing bilateral were excluded. NVB = neurovascular bundle.

Of those 28 patients with ≥rT3 and primary NSS harbouring PSM, the area of PSM was unrelated to NSS with IFST in 85.7% (*n* = 24) of the patients (area of ductus deferens in 14.3% (*n* = 4), area of secondary resected NVB in 25% (*n* = 7), area outside of NVB in 14.8% (*n* = 4), area outside of primary nerve resection and unilateral NSS in 21.4% (*n* = 6) and pT4 in 10.7% (*n* = 3)). In 10.7% (*n* = 3) of men, the reason for PSM was possibly related to NSS with IFST, as PSM was in the area of the NVB but IFST and final pathology of the specimen of IFST showed NSM. In 3.6% (*n* = 1) of patients, the area of PSM was related to initial NSM in IFST (false-negative IFST), as the final pathology results of IFST showed PSM (Figure 1).

## 4. Discussion

This large prospective series of 126 consecutive clinical high-risk PCa patients undergoing RP demonstrated that the prediction of tumour stage through preoperative mpMRI is essential but must be considered carefully—especially when this information is used to decide whether NSS will be performed. IFST and other techniques should be additionally used to achieve higher rates of nerve-sparing procedures with lower PSM and to improve functional outcomes [19,21,22]. Our analyses revealed some noteworthy results.

First, of all 83 patients with locally advanced disease (pT3-4), ECE was preoperatively detected in 54 of these patients (65.1%). In other words, 34.9% (*n* = 29) with locally advanced disease (pT3–4) were understaged by preoperative mpMRI which can result in higher PSM rates when the decision to undergo NSS is solely based on preoperative mpMRI. Moreover, 27.9% (*n* = 12) of patients that harboured pT2 in the specimen were overstaged to ECE by preoperative mpMRI, which might have led to primary nerve resection, when only relying on mpMRI as a decision-making tool for NSS. These results led to a sensitivity of 65.1% and specificity of 72.1% for detecting tumour stage ≥pT3, with a positive predictive value of 81.8%. Comparing these findings with data reported in the literature, where most of the studies show that more than half of the cases with locally advanced cancer (pT3–4) were not detected in preoperative mpMRI, our results seem to be slightly better [17,23]. In a meta-analysis by Jansen et al., the sensitivity of mpMRI for overall stage T3 detection in all tumour stages of PCa was only 61% [23]. However, when comparing our data to these studies it should be mentioned that our study exclusively included patients with high-risk PCa who have a higher risk of ECE which might influence concordant rates. In conclusion, an mpMRI is essential in patients with high-risk PCa for disease management and when choosing the surgical pathway providing information for surgeons and patients of the probability of a multimodal path including adjuvant radiotherapy. Intraoperatively, however, surgeons should be careful when using only preoperative mpMRI findings in surgical planning.

Second, by only using preoperative nomograms, the mean risk of ECE was predicted to be only 71.8% for subsequent ≥pT3, whereas the mean risk of ECE was predicted to be 60.1% for pT2. When ECE was diagnosed in mpMRI, nomogram predicted the risk for ≥pT3 to be 72.8%. Consequently, the sole use of nomograms to predict ECE cannot be recommended either. Interestingly, a study by Lebacle et al. showed that the integration of MRI with clinical data for predicting the pathological stage before RP permitted them to accurately exclude ECE in 74% of cases [24]. Considering these findings, a combination of nomograms and mpMRI could be a suitable way to preoperatively predict ECE.

Third, in our analysis, 50.0% (*n* = 28) of men with ≥ rT3 stage had a negative surgical margin in addition to a nerve-sparing procedure using IFST. Using IFST and after secondary resection, bilateral, unilateral and no nerve sparing were performed in ≥rT3 patients, 42.9% (*n* = 24), 51.8% (*n* = 29) and 5.3% (*n* = 3). Without IFST and relying just on the results of preoperative mpMRI, at least some of these patients with ≥rT3 would not have undergone NSS, possibly resulting in worse functional outcomes such as erectile dysfunction and urinary incontinence [5]. Looking at PSM in patients with ≥rT3 primarily undergoing NSS, 50.0% (*n* = 28) of the patients undergoing NSS with IFST showed PSM in their final pathology. As the area where the NVB contacts the prostate is routinely checked by IFST and IFST has a low rate of false-negative results, most of the PSM in patients undergoing IFST are not directly related to nerve-sparing procedure [19,25,26]. Looking more closely at the area of PSM in these 28 patients, we considered 85.7% (*n* = 24) of patients to be unrelated to IFST and NSS, whereas 10.7% (*n* = 3) remained possibly caused by NSS with IFST and 3.6% (*n* = 1) was caused by NSS with false-negative IFST and might not be present, if all patients with ≥rT3 directly underwent bilateral resection of NVB. One must take into consideration that PSM is not always directly associated with biochemical recurrence (BCR) or disease progression. With a median follow-up of 48 months, Karl et al. showed that BCR only occurred in 39.7% of patients with at least pT3a and PSM [27]. As seen in several studies, IFST generally significantly increases nerve-sparing frequencies and is associated with lower PSM rates, especially in patients with pT3a/b [19,21]. This could also be proven for patients from our institution [16]. Moreover, at 80%, PSM in patients without NSS and IFST was higher in our cohort. Therefore, one can conclude that the additional use of IFST in patients with ≥rT3 PCa might further help decision making for or against nerve-sparing surgery, even for high-risk patients and one should not only rely on the information acquired by mpMRI.

There are several limitations of our study. First, the inherited limitations of a retrospective analysis of the data are to be mentioned. Nevertheless, all data were collected prospectively. Second, the mpMRIs were interpreted by different radiologists as the mpMRI were made at different institutions. A concern regarding mpMRI is the considerable interobserver variability, as the experience of the reader remains of paramount importance [28,29]. However, the study of Jäderling et al. showed that combining preoperative prostate MRI with a preoperative MRI conference affects the effectiveness of nerve-sparing surgery and reduces positive surgical margins [30]. Therefore, especially for patients with high-risk disease the radiological examinations should be able to be revised by a dedicated radiologist in a multidisciplinary context. Third, we were not able to determine, on which parameters the decision to primarily not undergo NSS in 15 patients (11.9%) was based. Fourth, the classification of PSM in patients undergoing IFST to be related or unrelated to IFST was solely based on the written pathologic report and leaves space for interpretation. Finally, the correlation of the exact localization of radiologic extraprostatic tumour spread with PSM in final pathology was not possible.

## 5. Conclusions

mpMRI in patients with high-risk PCa provides essential information on the local tumour spread. Nevertheless, our results indicate that especially sensitivity and NPV for detecting ECE using preoperative mpMRI is low. Therefore, one should not solely rely on mpMRI or the use of a nomogram to predict ECE and decide whether NSS is performed for patients with high-risk PCa, as some patients can safely undergo NSS as represented by the use of IFST even if ECE is suspected in mpMRI.

## Figures and Tables

**Table 1:** Descriptive characteristics of 126 consecutive patients with high-risk prostate cancer undergoing RP at the University Hospital Frankfurt between 01/2018 and 06/2020 & cross-tabulation of radiological tumour stage (rT) and pathological tumour stage (pT) for 126 patients undergoing preoperative mpMRI of the prostate and RP.

Variable				Patients				
BMI, kg/m^2^, median (IQR)				26.5 (24.7–29.9)				
Age at RP, yrs, median (IQR)				66.0 (60.5–72)				
Age, *n* (%)								
<60				22 (17.7)				
60–70				62 (49.0)				
71–79				42 (33.3)				
iPSA (ng/ml), median (IQR)				10.0 (6.0–20.7)				
iPSA, *n* (%)								
<20 ng/ml				96 (76.2)				
≥20 ng/ml				30 (23.8)				
DRE, *n* (%)								
<cT2c				84 (66.7)				
≥cT2c				42 (33.3)				
Biopsy Gleason-Score, *n* (%)								
<8				27 (21.4)				
≥8				99 (78.6)				
ISUP specimen, *n* (%)								
1				3 (2.4)				
2				12 (9.5)				
3				12 (9.5)				
4				59 (46.8)				
5				40 (31.8)				
Surgical technique, *n* (%)								
Open				86 (68.3)				
Robotic-assisted				40 (31.7)				
rT; *n* (%)								
rT2				60 (47.6)				
rT3a				38 (30.1)				
rT3b				27 (21.4)				
rT4				1 (0.9)				
rN; *n* (%)								
rN0				111 (88.1)				
rN1				15 (11.9)				
pT; *n* (%)								
pT2				43 (34.1)				
pT3a				49 (38.9)				
pT3b				30 (23.8)				
pT4				4 (3.2)				
pN; *n* (%)								
pN0				99 (78.6)				
pN1				25 (19.8)				
pNX				2 (1.6)				
		Pathological tumour stage						
		pT2	pT3				pT4	Total
			Total		pT3a	pT3b		
Radiological tumour stage								
rT2		31	28		21	7	1	60
rT3	Total	12	50		27	23	3	65
	rT3a	10	26		17	9	2	38
	rT3b	2	24		10	14	1	27
rT4		0	1		1	0	0	1
Total		43	79		49	30	4	126 100%

Abbreviation register: BMI:Body Mass Index; iPSA: intial PSA; RP: radical prostatectomy; yrs: years; IQR: inter-quartile range; DRE: digital rectal examination, rT: radiologic stage; pT: pathologic stage.

**Table 2:** Sensitivity analysis for detection of extraprostatic disease (≥T3) with preoperative mpMRI of the prostate.

**mpMRI** ***n*** **= 126**	**Reference: p ≥ T3** **according Imaging**		
	p ≤ T2 *n* = 43 (34.1%)	p ≥ T3 *n* = 83 (65.9%)	
r ≤ 2 *n* = 60 (47.6%)	TN 31 (51.7%)	FN 29 (48.3%)	NPV =TN/(FN + TN) 51.7%
r ≥ T3 *n* = 66 (52.4%)	FP 12 (18.2%)	TP 54 (81.8%)	PPV =TP/(TP + FP) 81.8%
	Specificity =TN/(FP + TN) 72.1%	Sensitivity =TP/(TP + FN) 65.1%	Accuracy =(TP + TN)/all 67.5%

Abbreviation register: TN—total negative; FN—false negative; FP—false positive; TP—total positive; NPV: negative predictive value; PPV: positive predictive value; pT: pathological stage; rT: radiologic stage.

**Table 3:** Summary of nerve-sparing procedures for high-risk patients with PSM outcome.

		Primary Nerve-Sparing			Final Nerve-Sparing (including Secondary Resection in Case of Positive IFST)			PSM-Total (with IFST)	
	*n*	Bilateral	Unilateral	none	Bilateral	Unilateral	none	no	yes
rT2	60	54	1	5	41	13	6	45 (44)	15 (11)
rT3a	38	33	2	3	17	17	4	18 (17)	20 (19)
rT3b	27	14	6	7	7	11	9	12 (11)	15 (9)
rT4	1	1	0	0	0	1	0	0 (0)	1 (1)
≥rT3	66	48 72.7%	8 12.1%	10 15.2%	24 42.9%	29 51.8%	13 5.3%	30 (28) 50%	36 (28) 50%

Patients with partial nerve resection were also counted as unilateral secondary nerve-sparing (rT2 *n* = 4; rT3a *n* = 4; rT3b *n* = 1). Percentage from ≥rT3: total patients (*n* = 66) with primary nerve-sparing from all patients; indicated in bold are cases of bilateral, unilateral or no nerve-sparing and PSM rate after secondary resection counted from patients with primary nerve-sparing (*n* = 56).

## Data Availability

All datasets generated for this study are included in the manuscript. C.H. had full access to all the data in the study and takes responsibility for the integrity of the data and the accuracy of the data analysis.
